# Clinical and Radiological Follow-Up of a Pfizer-BioNTech COVID-19 Vaccine-Induced Hemichorea-Hemiballismus; Insights Into Mechanisms of Basal Ganglia Dysfunction

**DOI:** 10.5334/tohm.697

**Published:** 2022-05-18

**Authors:** Molly Cincotta, Ruth H. Walker

**Affiliations:** 1 University of Pennsylvania, US; 2 Parkinson’s Disease and Movement Disorder Center, US; 3 Department of Neurology, James J. Peters Veterans Affairs Medical Center, US; 4 Department of Neurology, Mount Sinai School of Medicine, US

**Keywords:** chorea, hemiballismus, hemichorea, COVID-19, vaccination

## Abstract

Asymmetric chorea unrelated to structural lesions is typically due to systemic etiologies, such as metabolic, autoimmune, or other inflammatory disorders. This is an editorial commenting on a paper by Batot C, Chea M, Zeidan S, et al. Clinical and radiological follow up of Pfizer-BioNTech COVID-19 vaccine-induced hemichorea-hemiballismus. *Tremor and Other Hyperkinetic Movements*; 2022; 12(1). DOI: *https://doi.org/10.5334/tohm.688*. A 90-year-old patient is reported who developed hemichorea shortly after his second vaccination against COVID-19. Hypometabolism was noted in the contralateral striatum. This case provides potential insights and raises questions about mechanisms of immune-mediated hemichorea.

## Introduction

Batot et al [1] report a 90-year-old patient with vascular risk favors including high blood pressure, atrial fibrillation and prior myocardial infarction, who developed left-sided hemichorea within hours of his second dose of Pfizer Bio-N-Tech SARS-COVID2 vaccination and 21 days following his initial vaccination. The evaluation notably revealed intrathecal immunoglobulin synthesis of oligoclonal bands (OCBs) and decreased uptake in the right putamen on [18F]-fluorodeoxyglucose-positron emission tomography (FDG-PET.) The patient’s symptoms and the abnormal PET responded favorably to corticosteroid treatment, arguing for an autoimmune or inflammatory etiology for his symptoms [1].

This case is an interesting addition to the scientific literature and invites further questions about mechanism and treatment of immune-mediated hemichorea.

## Discussion

Although the most common causes of hemichorea/hemiballismus are ischemic or vascular in nature, as would be expected for such a focal neurologic phenomenology, systemic etiologies are surprisingly common [2]. The most frequently reported cause in adults is hyperglycemic hemichorea/hemiballismus (HC/HB), which generally occurs in older patients with longstanding type II diabetes mellitus in non-ketotic diabetic crisis; the pathophysiology responsible for the generation of the movement disorder in this situation is not well understood. The most well-known cause of asymmetric or hemichorea attributable to a presumed autoimmune response is, of course, Sydenham’s disease, which occurs primarily in children. Other inflammatory/auto-immune causes of hemichorea include antiphospholipid syndrome (APLS) with or without systemic lupus erythematosus (SLE) and autoimmune encephalitides such as anti-N-methyl-D-aspartate (NMDA) receptor encephalitis [2]. Additionally, a history of inflammatory chorea appears to predispose toward recurrence later in life, as can be seen in women recovered from Sydenham’s disease in childhood who go on to develop estrogen-induced chorea or chorea gravidarum in adulthood [2]. Despite these well-described entities, the mechanisms responsible for the appearance of chorea and the reasons for the asymmetry are not well understood [2].

Chorea does not appear to be a common symptom of acute COVID-19 infection. A prospective cohort in the United Kingdom reported that 2 of 1,334 children and adolescents hospitalized with acute COVID-19 infection manifested chorea [3]. In a recent review of movement disorders seen in acute COVID-19 infections, non-drug-induced choreiform movements were only reported in one case, with myoclonus and tremor predominating among the hyperkinetic presentations [4].

A literature search found only one case of vaccination-induced chorea prior to the current pandemic. This case was in an 11-year-old girl who developed generalized chorea after receiving vaccination against human papilloma virus [5]. In context of the current pandemic and widespread vaccination, chorea appears to be a remarkably rare complication, however the authors note that it has been reported in other patients receiving COVID-19 vaccination; one case also receiving the Pfizer vaccine [6] and two others receiving AstraZeneca AZD1222 [7]. All cases were unilateral in nature. They also report that according to Vigilyze pharmacovigilance database, around 40 cases of hemichorea/hemiballismus have been reported which were attributed to the Pfizer Bio-N-Tech vaccination, although the details of these cases are unknown [1].

Similar to these reported cases, Batot et al.’s patient was elderly; the other patients were 83, 84 and 88 at the time of presentation [1,6,7], although the authors do note this could be in part due to focused efforts to vaccinate the elderly at-risk population early after the vaccine’s release. Metabolic hemichorea, such as hyperglycemia-related HC/HB, is predominantly seen in elderly patients, thus raising the question that age-related factors such as asymmetric, subclinical neurodegeneration or vascular lesions could play a role in both predisposition to such conditions, and in their asymmetry.

The timing of onset of symptoms in the current case supports an immune-mediated mechanism. Initial studies of the immune response to the Pfizer vaccine demonstrated that antibody titers peak after 14 days on average following the initial injection, with a subsequent relative plateau [8]. Secondary injection 28 days later results in a brisker immune response with an immediate increase in antibodies [8]. This would be consistent with the timing in the current case, with the movement disorder developing at 21 days after the initial injection and hours after the second dose [1]. The other case of hemichorea secondary to Pfizer-BioNTech vaccination occurred at about one day following the second inoculation [6]. Other case reports of hemichorea were following the AstraZeneca AZD1222 vaccine and occurred at 16 and 40 days after the first dose [7]. There is no mention of a second injection for those individuals, although the recommended interval between the two doses of this vaccine is 11–12 weeks suggesting they were not yet due for the second inoculation.

Cerebrospinal fluid (CSF) analysis in the current patient demonstrated elevated oligoclonal bands with an otherwise normal profile [1]. CSF analyses in movement disorders due to COVID-19 infection have largely been normal or nonspecific [4]. Abnormalities include a mild to moderate increase in protein and white count; OCBs were only positive in one case of diplopia, ataxia and weakness [9]; one patient with encephalopathy with chorea had elevated myelin basic protein [4]. Of the vaccine-induced chorea cases, no CSF analysis was performed on the patient who received the Pfizer-BioNTech vaccination [6]; the two who received AstraZeneca AZD 1222 showed only elevated protein [7]. OCBs were not examined in either case. OCBs were not detected in the case of HPV vaccination-induced hemichorea [5]. These findings are non-specific, but are consistent with mild central nervous system inflammation.

As we have discussed [2], the pathophysiology in systemic causes of hemichorea is not well understood, nor is the reason for the asymmetric presentation. Some theories posit underlying asymmetry in microvascular structure, either due to atherosclerosis in the case of non-ketotic hyperglycemia, or small vessel vasculitis in the case of inflammatory disorders. It is also possible that there is lateral dominance of the deep grey structures analogous to that found in the cortex, although this theory does not have much data to support it [10].

Advanced imaging has been frequently used to help better clarify the underlying changes in non-lesional hemichorea. In this case report, the authors note reversible findings on FDG-PET, with initial decrease in contralateral putamen uptake while the patient was symptomatic that resolved following steroid treatment [1]. PET findings in hemichorea can vary, and the underlying etiology likely determines the direction of the change [11]. The results in the current case are in contrast to the typical findings in inflammatory/infectious hemichorea, in which there is generally either normal uptake or hypermetabolism contralateral to the lesion [12,13,14,15]. In hyperglycemic HC/HB report reduced uptake is more common [2,16], although even in this situation there can be variability, with one report of two patients showing opposing results [17]. We hope that developments in advanced imaging will eventually shed light on the underlying mechanisms responsible for the variability of these observations, and will give meaningful clues to etiology and management.

In conclusion, the case report presented by Batot et al. adds a new etiology of acquired, non-lesional hemichorea to the literature. While the side effect appears to be rare, it is an important consideration in the current COVID-19 era, as the appropriate treatment is corticosteroids or potentially other immune-modifying medications, rather than the dopamine receptor-blocking medications traditionally used in the symptomatic management of chorea. In addition, the reports of this side effect due to vaccinations rather than in the setting of acute infection raises interesting questions about the mechanism by which such dysimmune reactions are triggered. Finally, although an autoimmune etiology is strongly implicated in the reported case, and it would be expected that the mechanism would be similar to that of other inflammatory causes of hemichorea, the FDG-PET findings seen here resembled those seen in hyperglycemic HC/HB, and might indicate a shared mechanism at some level.
